# 571 Evaluating the Relationship Between BMI and Frailty in Older Adult Burn Patients

**DOI:** 10.1093/jbcr/iraf019.200

**Published:** 2025-04-01

**Authors:** Andrew Bieterman, Amanda Soo Ping Chow, Caitlin Mehta, Dhaval Bhavsar, David Hill, Sara Higginson, Theresa Chin, Kathleen Romanowski, Lauren Nosanov, Sam Miotke, Colette Galet, Tuan Le, Melissa McLawhorn, Lauren Moffatt, Taryn Travis, Jeffrey Shupp, Shawn Tejiram

**Affiliations:** The Burn Center at MedStar Washington Hospital Center; The Burn Center at MedStar Washington Hospital Center; Georgetown University; The University of Kansas Health System Burnett Burn Center; Regional One Health; Shriners Children’s - Ohio; University of California Irvine; University of California, Davis and Shriners Children’s; Emory University School of Medicine; Regions Hospital; University Of Iowa; MedStar Health Research Institute; MedStar Health Research Institute; MedStar Health Research Institute; MedStar Washington Hospital Center; MedStar Washington Hospital Center; MedStar Washington Hospital Center

## Abstract

**Introduction:**

Frailty refers to an age-related syndrome of functional and physiological decline that is characterized by heightened vulnerability to adverse health events. Previous literature has demonstrated the efficacy of frailty assessment among burn injured patients. There is a U-shaped association between body mass index (BMI) and frailty with both ends of the BMI spectrum representing higher risk for elevated frailty scores. BMI has previously been shown to have a significant impact on inpatient length of stay, adverse events, and mortality. Despite this, there is a paucity of literature evaluating the complex relationship between frailty and BMI in burn patients. In this study, we investigated the relationship between BMI and frailty scores and their effects on burn outcomes in a multicenter population of older adult burn patients.

**Methods:**

Burn injured patients admitted to 12 burn centers from January 2017 to December 2019 who were 60 years and older were retrospectively reviewed. Demographics, injury characteristics, and clinical metrics were obtained. Frailty was assigned to patients using the Canadian Study of Health and Aging Clinical Frailty Scale (CSHA-CFS). BMI was used to stratify patients as underweight (UW; BMI< 18.5), non-obese (NO; BMI 18.5-24.9), overweight (OW; BMI 25-29.9), obese I (OI; BMI 30-34.9), obese II (OII; BMI 35-39.9), or obese III (OIII; BMI≥40). Outcomes evaluated included the number of operations per admission, length of stay (LOS), and in-hospital mortality. Data is presented as mean ± SD. Frailty score by BMI, LOS and mortality were examined by Kruskal-Wallis test.

**Results:**

Of 1,632 older adult burn patients, 1,415 patients had BMI data and were included for study. Of these, 49 were UW, 413 NO, 475 OW, 278 OI, 105 OII, and 95 OIII. Mean age was 70±8.5 years, mean TBSA burn was 8.4±12.1%, and the mortality rate was 8.8%. Frailty score was higher in UW (4.7±1.1) compared to NW (4.1±1.4; p=0.03), OW (3.8±1.3; p< 0.0001), and OI (3.8±1.2; p=0.0001). Frailty in NW (4.1±1.4) was also higher compared to OW (3.8±1.3; p=0.01) and OI (3.8±1.2; p=0.04). Total operations, LOS, and mortality were not significantly affected by BMI classification.

**Conclusions:**

Frailty scores were significantly higher in underweight patients compared to normal weight, overweight or obese burn patients. Differences in BMI did not affect the LOS, total operations or mortality.

**Applicability of Research to Practice:**

Although the relationship of BMI and frailty remain complex, understanding the effect of BMI extremes like being underweight may help with determining frailty status.

**Funding for the Study:**

N/A

